# Development of novel *Streptococcus equi* vaccines with an assessment of their immunizing potentials and protective efficacies

**DOI:** 10.1186/s12917-024-04012-z

**Published:** 2024-05-03

**Authors:** Rafik Soliman, Mohamed Yousef, Sara Abdel gelil, Hassan Aboul-Ella

**Affiliations:** 1 Department of Microbiology, Faculty of Veterinary Medicine, Cairo University, Giza, Egypt; 2 Department of Veterinary Hygiene, Faculty of Veterinary Medicine, Cairo University, Giza, Egypt

**Keywords:** Strangles, *Streptococcus equi*, *Streptococcus zooepidemicus*, Novel combined vaccine, MONTANIDE adjuvants

## Abstract

Strangles is a highly contagious disease of the equine upper respiratory tract caused by *Streptococcus equi* subspecies. *Streptococcus equi* subsp. *equi* (*S. equi*) and *Streptococcus equi* subsp. *zooepidemicus* (*S. zooepidemicus*) was isolated, as local, hot, and field strains, from horses clinically suffering from respiratory distress. The isolated *Streptococci* were identified using bacteriological and molecular techniques. Four formulations of inactivated *S. equi* vaccines were developed and evaluated. The first formulation was prepared using the *S. equi* isolates, adjuvanted with MONTANIDE GEL adjuvant, while the second formulation was adjuvanted with MONTANIDE ISA-70 adjuvant. The other 2 formulations were inactivated combined vaccines prepared from both *S. equi* and *S. zooepidemicus* isolates. The 3rd formulation was the combined isolates adjuvanted with MONTANIDE GEL while the 4th formulation was the combined isolates adjuvanted with MONTANIDE ISA-70. The developed vaccines’ physical properties, purity, sterility, safety, and potency were ensured. The immunizing efficacy was determined in isogenic BALB/c mice and white New Zealand rabbits using the passive hemagglutination test. Also, the antibodies’ titer of the combined *S. equi* and *S. zooepidemicus* vaccine adjuvanted with MONTANIDE ISA-70 in foals was tracked using an indirect enzyme-linked immunosorbent assay. The protective efficacy of the developed vaccines was determined using a challenge test in both laboratory and field animal models, where a 75% protection rate was achieved. The combined vaccine proved to be more efficacious than the monovalent vaccine. Also, the MONTANIDE ISA-70 adjuvant provided significant protective efficacy than the MONTANIDE GEL. The current work is introducing a very promising mitigative and strategic controlling solution for strangles.

## Introduction

The control of many important equine infectious diseases remains challenging and strangles is one of these infections that affect horses, donkeys, and mules at any age [1]. Strangles caused by *S. equi* is one of the most widespread and costly horse diseases that led to devastating epidemics in stables where horses are housed [2, 3]. It is an acute, contagious, and deadly respiratory tract infection. The typical signs include pyrexia, suppurative mucopurulent nasal discharge, lymphadenitis, and abscess formation often in the head and neck lymph nodes. Other lymph nodes and organs can be affected, resulting in a severe stage of the disease called “bastard strangles” [4].

*S. equi* infection produces high morbidity and low mortality in susceptible populations previously free of disease and the transmission of the disease occurs via direct contact with infectious exudates and indirectly through fomite transmission [2, 5]. The disease causes major economic losses to the equine industry worldwide due to its prolonged course, extended recovery period, and associated serious complications.

*S. equi* persists alive in the contaminated environment only for 3–4 weeks and the infected horses stand behind the persistence and spread of the infection. Even if the infected animals recover from the disease, 10% of these animals remain as long-term *S. equi* carriers, harboring the microorganism for months. In these long-term carriers, the presence of the pathogen is not detectable, and the carrier animals do not show any clinical signs of disease [4].

*S. zooepidemicus*, on the other hand, causes mild cases of respiratory affection [6] and can be isolated from horses with confirmed *S. equi* infection [7]. Although most horses are colonized normally by the *S. zooepidemicus*, which shares many cross-reactive immunogenic proteins with the clonal *S. equi*, these horses are not protected against strangles. Conversely, strangles vaccines prepared from *S. equi* do not protect against respiratory or uterine infections caused by *S. zooepidemicus*.

Prevention of strangles through quarantine and screening is particularly difficult where there is frequent movement and mixing of horses during the breeding season and at racetracks, where strangles outbreaks have not been appropriately investigated and controlled. On the other hand, the use of antibiotics has sparked much controversy. Antibiotics can be counter-productive, especially with the emergence of antibiotic-resistant *S. equi* [8, 9]. Antibiotics suppress bacteria for a time, but infection may flare up when the antibiotics are discontinued. Antibiotic-treated horses might become re-infected because they do not develop protective immunity [10] as well as the use of some antibiotics becomes ineffective when external signs of disease develop [11].

Different types of *Streptococcus* vaccines have been developed and applied for the control of this disease in equines. These include killed, cell extract vaccines [12], live-attenuated vaccines [13], and other forms of the live-attenuated vaccine, which is the Pinnacle IN (ZOETIS, USA) that is available in North America and some other countries for intranasal administration. A subunit vaccine prepared from recombinant *Streptococcus equi* subsp. *equi* (SEE) proteins have also been produced and applied to control this infection. Most of these vaccines, however, conferred limited protection [1]. On the cusp of the immunization era, active and passive immunization strategies are considered the cornerstone of a wide range of a variety of significant infectious diseases’ prophylactic, therapeutic, and diagnostic approaches [14–21], in this context the primary objective of the current investigation was to develop four novel *S. equi* vaccines and assess each vaccine’s ability to immunize and protect using various animal models, through the introduction of those newly developed inactivated *Streptococcus* vaccines empowered by strong adjuvants and prepared from *S. equi* alone or combined with *S. zooepidemicus* with a complete evaluation of their immunizing potentials and protective efficacies.

## Materials and methods

### Laboratory animals’ source and fate

All laboratory animals involved in the current study were purchased from a commercial private laboratory animal farm in El-Fayoum governorate, Egypt, and hosted by the laboratory animal unit at the Faculty of Veterinary Medicine, Cairo University. Laboratory animals that died during the study were bio-safely disposed and the rest of the laboratory animals that remained alive after the end of the experiment were neither released nor euthanized, they are kept, and properly managed till being reused, reassigned in other experiments, or naturally died, and then bio-safely disposed of.

### Sampling from clinically diagnosed active strangles cases

Samples were collected from horses with clinical signs of upper respiratory tract infections. These horses were from stables with confirmed *S. equi* infection and displayed one or more clinical signs of strangles. The samples included pus from ripened abscesses of the submandibular lymph nodes in 1-year-old horses and nasal swabs from nasal cavities of diseased horses taken from a distant part of the nasal cavities after careful cleaning of nares and anterior part of the nasal mucosa. The cleaning was carried out with water, soap, and betadine antiseptic. All sampling procedures were carried out through a standardized protocol [22], by the hand of an expert equine veterinarian. All the samples were collected in duplicate and processed for bacterial isolation in a duration ranging from 0.5 to 4 h after collection.

### Isolation and phenotypic identification of the *S. equi* and *S. zooepidemicus*

The collected samples were streaked into Edward’s modified medium with colistin sulfate (5 mg/l) and oxolinic acid (2.5 mg/l) (OXOID, UK). This culture medium showed the highest sensitivity (100%) and specificity (100%) for streptococci, and it is a selective media for primary isolation [23]. The inoculated plates were incubated in 5% (v/v) CO_2_ at 37ºC for 24–48 h. Typical β hemolytic streptococci dew pinpointed-like colonies developed on the inoculated plates and were identified by the characteristic colony morphology, Gram’s technique, and biochemical testing including catalase test confirmed the isolates. Recovered isolates identified as *S. equi* fermented sucrose and salicin but not lactose, sorbitol, or trehalose, while isolates identified as *S. zooepidemicus* developed the same biochemical results but fermented lactose and sorbitol [24, 25].

### Molecular confirmation of the recovered *S. equi* subspecies using polymerase chain reaction (PCR)

The phenotypically identified isolates were confirmed with PCR, which was designed to detect the DNA sequence of *seM*, the gene for the anti-phagocytic M protein of *S. equi*, using one sequence of primer *seM* [26, 27] and *sodA* gene for *S. zooepidemicus* [28].

The DNA extraction and the two separate PCR primers used for identification of the recovered isolates were based on previous studies [29–31] with some modifications in Table 1.

**Table 1:** Primer sequences for *seM* and *sodA* genes amplification

Primer name	Primer sequence (5’-3’)	Annealing Temperature	Product Size	Reference
***seM***	TGCATAAAGAAGTTCCTGTC **(F)** GATTCGGTAAGACCTTGACG **(R)**	72ºC	677 bp	[26]
***sodA***	CAGCATTCCTGCTGACATTCGTCAGG **(F)** CTGACCAGCCTTATTCACAACCAGCC **(R)**	56ºC	235 bp	[28]

The *seM* program: included a denaturation step of 94 °C for 2 min and then subjected to 35 cycles of amplification, each consisting of (denaturation at 94 °C for 10 s, annealing at 70 °C for 10 s, and extension at 72˚C for 5 s, which was followed by a final extension at 72 °C for 5 min). The *sodA* program was done under the same conditions as the *seM* program, except for the annealing temperature, which was kept at 56˚C. The expected bands of amplicons were 235 bp in *S. zooepidemicus* and 677 bp *in S. equi*. The PCR products were visualized using gel electrophoresis in a 2% gel at 80 V.

### Bacterial propagation, inactivation, concentration, antigen preparation, and vaccine formulation

One liter of an 18-hours-old broth culture of *S. equi* was inoculated into nine liters of previously warmed Todd-Hewitt broth (OXOID, UK) with 10% horse serum, then incubated at 37 °C. After six hours, the bacterial cells were separated by centrifugation at 7000xg for one hour, using a cooling centrifuge (JOUAN, France), washed twice with sterile normal saline, and finally with phosphate-buffered saline (PBS) pH 7.0. The washed cells were suspended in normal saline at a concentration of 1 × 10^10^ colony-forming units/ml (CFU/ml) based on the total bacterial count (TBC) test. The bacterial cell suspension was distributed in two equal volumes as follows. The cell concentration in the first part was adjusted to 2.5 × 10^9^ CFU/ml based on the total bacterial count (TBC) test and inactivated by adding 0.2% formalin and kept undisturbed for 18 h at room temperature (25 °C). MONTANIDE GEL adjuvant (SEPPIC, France) was added to the inactivated *S. equi* culture in a ratio of 10 (adjuvant):90 (bacterial cells suspension), while the MONTANIDE ISA-70 (SEPPIC, France) was used in a ratio of 70 (adjuvant):30 (bacterial cells suspension). The same procedures were used for the preparation of the other vaccine formulation using the *S. zooepidemicus* strain. The bacterial cell suspension part of the four developed formulations was the same equal to 2.5 × 10^9^ CFU/ml.

Four formulations of inactivated *S. equi* subspecies vaccines were prepared using the recovered field isolates as master seeds. These include *S. equi* adjuvanted with MONTANIDE GEL, *S. equi* adjuvanted with MONTANIDE ISA-70, combined *S. equi and S. zooepidemicus* adjuvanted with MONTANIDE GEL, and combined *S. equi* and *S. zooepidemicus* adjuvanted with MONTANIDE ISA-70.

### Physical properties and the basic quality control characteristics of the developed and prepared vaccines

#### Physical properties of the developed and prepared vaccines

The physical properties of vaccine emulsions were investigated for determination of the emulsion type, stability, and formalin residues [18].

#### Emulsion type

Using a drop test, 2 drops of emulsion were placed separately on a clean glass microscopic slide and each drop was mixed with either one drop of oil or one drop of water. A water-in-oil emulsion drop blends easily and readily with the oil drop, but the two phases will be separated when mixed with the water drop and vice versa in the case of the oil-in-water emulsion.

#### Emulsion stability

Short-term stability: was evaluated by dropping a sample from each formulation in a beaker of distilled water and kept overnight at 6–8 °C stable emulsion will keep in its white color and heavy creamy consistency while unstable emulsion will be separated into two watery and oily drops.

long-term stability: was evaluated by keeping retention samples from each formulation at the recommended storage temperature (6–8 °C) and regularly observing those samples, stable emulsion will keep its consistency for a longer storage period (months and years) while unstable emulsion will be separated into watery and oily phases in shorter storage period (days and weeks).

#### Formalin residues

The determination of formalin residue was done using the phenyl hydrazine method according to the Egyptian Veterinary Codex 2009, (CLEVB). Briefly, 1 ml of standard formaldehyde solutions were freshly prepared by adding aqueous formaldehyde solution (40%) to distilled water in five test tubes representing five concentrations (1.0, 0.50, 0.1, 0.05, and 0.01%) of the formaldehyde standards. In another test tube represented the sample (1 ml of the tested inactivated vaccine added to 99 ml distilled water to achieve 1% concentration), the following reagents were put in order: 0.1 ml of phenyl hydrazine 1% (Riedel-de Haen Allied Signal, Germany), 0.1 ml of potassium ferricyanide 5% (BDH Chemicals Ltd., Poole, England) and three drops (90 µl) of concentrated hydrochloric acid (HCl) (The Egyptian Company for Chemicals and Drugs (ADWIA), Egypt). This mixture was thoroughly shaken and placed on the bench for 5 min before the color of the sample tube and the standard tubes were matched.

### Basic quality controls (QCs) characteristics of the developed and prepared vaccines

Tests for purity, sterility, and safety were performed according to [32, 33], repeated in triplicates for each test and for three different samples from each newly developed vaccine formulation.

#### Purity testing

Retention samples of the final completed product from each vaccine preparation were tested for viable bacteria and fungi as follows: One ml from each sample was inoculated into corresponding individual test bottles of culture medium (Brain heart infusion (BHI) broth for bacteria and Sabouraud’s dextrose broth (SDB) for fungi). The inoculated bottles were incubated for an observation period of 14 days at 37 °C to test for bacterial growth and 14 days at 25 °C to test for fungal growth. No growth is detected by the absence of turbidity in the case of bacteria and the absence of both turbidity and visible colonial fungal matt growth in the case of fungi, and microscopic examination of samples from all inoculated and incubated test bottles shows the typical microscopic characteristics of the involved bacterial strains indicates that the produced vaccine met the requirements of the test.

#### Sterility testing

Retention samples of the final completed product from each vaccine preparation were tested for the absence of viable bacteria and fungi were made by culturing on blood agar plates. Under complete aseptic condition loopful from each tested vial was opened and loopful of the contained vaccine was quadrantally streaked on a blood agar plate. The streaked plates were incubated for an observation period of 14 days at 37 °C to test for bacterial growth and 14 days at 25 °C to test for fungal growth. For the vaccine to pass this test, tests for sterility and freedom from contamination should show no growth of bacteria or fungi on blood agar.

#### Safety testing

Retention samples of the final completed product from each newly developed vaccine formulation were tested for safety in adult mice as follows: a 0.5 ml dose of each tested vaccine formulation was injected intraperitoneally into each of eight mice and two mice kept as non-vaccinated negative controls that were injected with 0.5 ml PBS as a placebo, and the animals observed for 7 days for any behavioral or physical (local or general) abnormal reactions. The safety of the newly developed vaccine formulations was co-evaluated in the field challenge in foals as described in the “field challenge in the unvaccinated/exposed foals’ section”. The preparation is considered unsatisfactory if adverse general or local reactions, attributable to the vaccine, occur in any of the mice (one week) and/or foals (one month) during the observation period.

### Immunizing potential and potency of the developed and prepared vaccines

#### Immunization of isogenic BALB/c mice

Thirty-two, 2-month-old isogenic BALB/c mice were randomly divided into four groups of eight mice each; five were vaccinated with the corresponding vaccine formulation and three were kept as a non-vaccinated negative control. The animals of each group were inoculated subcutaneously with 1/20 of a horse dose (0.25 ml) while the non-vaccinated negative controls were injected with 0.25 ml PBS as a placebo [24] of the respective vaccine formulation on days 0 and 14 of the experiment. Blood samples were collected from each animal before vaccination at days 0, 14, and 28 after applying the first dose of the vaccine.

#### Immunization of white New Zealand rabbits

Twelve adult male rabbits divided into four groups were used in this experiment. Each group consisted of 3 rabbits; two were vaccinated with the corresponding vaccine formulation and one was kept as a non-vaccinated negative control. The immunized rabbits of each group were inoculated intramuscularly three times, at ten-day intervals with 1 ml of respective *S. equi* vaccine formulation while the non-vaccinated negative controls were injected with 1 ml PBS as a placebo. Serum samples were collected the day before the vaccine injection (day 0) for the priming/initial dose and on days 7, 14, 21, 28, 35, 42, and 49. The collected serum was stored frozen at -20 °C until tested. The *S. equi*-specific antibody titers were determined using the passive hemagglutination (PHA) test. The reciprocal of the highest serum dilution showing a clear matt formation was considered the PHA antibody titer.

### Protective efficacy of the developed and prepared vaccines

#### Laboratory challenge of immunized BALB/c mice with *S. equi*

Fifteen mice were used per vaccine formulation to determine which induced the highest humoral immune responses (protective efficacy). In each group, twelve mice were injected intramuscularly with the newly developed vaccine formulation at the dose of 1 × 10^9^ CFU/ml while three mice were kept as non-vaccinated negative controls. A second booster dose was injected intramuscularly two weeks after the primary dose. Two weeks after the booster dose, each of the fifteen mice (per group of immunized and non-immunized mice) were challenged by intraperitoneal injection with 0.2 ml of viable *S. equi* bacterial suspension (1.25 × 10^6^ CFU/ml) according to [34]. The challenge inoculum was prepared from an 18-hour-old *S. equi* culture in trypticase soya broth, separately harvested, and re-suspended in PBS. The immunized and non-immunized mice were monitored daily for seven days following the challenge. The internal organs of the dead animals through the challenge stage were collected and aseptic bacterial isolation was performed to confirm the cause of death whether it was the challenged *S. equi* or not.

#### Field challenge in unvaccinated/exposed foals

Four foals, 2–3 months old were used for each vaccine formulation. Three out of the four were vaccinated while one was an unvaccinated negative control. The vaccination schedule consisted of two doses of 5 ml (2.5 × 10^9^ CFU/ml) of the vaccine formulation given intramuscularly at two weeks intervals while the non-vaccinated negative controls were injected with 5 ml PBS as a placebo. Serum samples were collected before immunization at day 0 and after immunization, at days 14 and 28, and stored frozen at -20 °C until testing.

### Humoral immune response tracking of the developed and prepared vaccines

#### Preparation of the partially purified M-protein from *S. equi (seM)*

Using the method of Woolcock [35, 36], a partially purified M-protein from *S. equi (seM)* was prepared and synthesized. Briefly, *S. equi* was cultured in Todd-Hewitt medium (OXOID, UK) for 24 h and centrifuged at 7000x for 5 min. The bacterial cells pellet was washed five times as follows; two times in normal saline, one time in distilled water, and two further washings in 0.1 M PBS, pH 7.0. The washed cells were re-suspended in normal saline, which was adjusted to pH 2.4 with 10 N HCl. The suspension was boiled at 95 °C for 12 min, then neutralized. The suspension was centrifuged at 7000x for 5 min and the supernatant was saved. The bacterial pellet was re-suspended in normal saline and the extraction procedure was repeated for an extra one more time. The supernatants were pooled and concentrated against polyvinylpyrrolidone (PVP). To 100 ml of the extract, 0.1 mg ribonuclease was added and dialyzed, using dialysis bags 12,000 molecular weight cut off (MWCO, SERVA, Germany), against 30 volumes of 0.01 M PBS, pH 8.0, at 37 °C for 5 h followed by further dialysis at 40 °C for 15–16 h. The dialyzed material was brought to 30% saturation with crystalline ammonium sulfate (OXOID, UK) at 40 °C and centrifuged at 20000x for 5 min using a cooling centrifuge (JOUAN, France). The precipitate was discarded, and the supernatant was brought to 60% saturation and re-centrifuged. The resultant precipitate was dissolved in sterile distilled water and freeze-dried. 12.5 g of the lyophilized material was dissolved in 50 ml of 0.1 M PBS, pH 7.4, and filter sterilized using a 0.22 μm Millipore filter. The resulting M protein is 325 mg/dl.

### Development of a homemade PHA and iELISA using the partially purified M-protein from *S. equi* (*seM*)

#### Passive hemagglutination assay (PHA)

The PHA was standardized for the rapid detection of anti- *S. equi* antibodies in sera of vaccinated animals according to [37].

#### Indirect enzyme-linked immunosorbent assay (iELISA)

A checkerboard titration was performed using different antigen concentrations and sera dilutions (a pool of sera from the day that supposedly would have the highest titer). The dilution that fell within the linear range of the iELISA was used. To detect specific antibodies against *S. equi*, individual sera were tested in triplicate. The 96 polystyrenes flat bottom ELISA plates were sensitized at 4ºC overnight with 50 µL of the prepared *seM* containing 12 µg/ml suspended in carbonate-bicarbonate buffer at pH 9.6. After this period, the plates were washed three times with PBS-T and blocked using phosphate-buffered saline-Tween 20 (PBST, SIGMA-ALDRICH, USA) with 5% nonfat milk at 37 °C, for 1 h. Sera of foals diluted 1:200 were added in duplicate to the wells and incubated for 1.5 h at 37ºC. After three washes with PBST, 1:10.000 peroxidase-conjugated anti-horse immunoglobulins G (IgG) (SIGMA-ALDRICH, USA) was added and incubated for 1.5 h at 37ºC. Ortho-phenyl diamine was used as chromogen and the OD read at 405 nm using the ELISA reader [38, 39].

### Statistical analysis

Data was organized into tables and figures using Microsoft Excel. A normality test was applied to determine whether the obtained data were parametric or non-parametric, using the Shapiro-Wilk test at a significance level of 0.05. Differences between time points were evaluated using Friedman’s test at a significance level of 0.05. Differences between groups of the nonparametric data were evaluated using the Kruskal-Wallis test. Differences between groups of the parametric data were evaluated using the t-test.

## Results

### Isolation and phenotypic identification of the *S. equi* and *S. zooepidemicus*

Five bacterial isolates were recovered from samples cultured on Edward’s blood agar plates. These isolates showed typical β-hemolytic colonies. Pure colonies stained by Gram’s stain and examined microscopically showed Gram-positive cocci that appeared in long chains. Biochemical examination of the isolates proved that all five isolates were catalase test negative. One isolate was negative for lactose and sorbitol fermentation and was identified as *S. equi* while four isolates were positive for lactose and sorbitol fermentation and were identified as *S. zooepidemicus* Table 2.

### Molecular confirmation of the recovered *S. equi* subspecies using polymerase chain reaction (PCR)

The expression products of *sodA* (235 bp), which are characteristic of *S. zooepidemicus* have been detected in 4/5 isolates. These four isolates gave negative results with the *seM* primers, while 1/5 isolates expressed the products of *seM* primers (677 bp), which is characteristic of *S. equi*.

**Table 2:** Results of *S. equi* isolation from diseased horses

Number of samples	Type of samples	Number of isolates	Phenotypic identification	Molecular confirmation
Six samples from 4 foals	Pus from a ripened abscessed submandibular lymph node.	4	*S. zooepidemicus*	*S. zooepidemicus*
One sample from a young horse	Nasal Swab.	1	*S. equi*	*S. equi*

### Bacterial propagation, inactivation, concentration, antigen preparation, and vaccine formulation

Four formulations of inactivated *S. equi* subspecies vaccines were prepared using the recovered field isolates as master seeds. These include *S. equi* adjuvanted with MONTANIDE GEL, *S. equi* adjuvanted with MONTANIDE ISA-70, combined *S. equi and S. zooepidemicus* adjuvanted with MONTANIDE GEL, and combined *S. equi* and *S. zooepidemicus* adjuvanted with MONTANIDE ISA-70.

### Physical properties and the basic quality control characteristics of the developed and prepared vaccines

#### Physical properties of the developed and prepared vaccines

***a- Emulsion type***.

The tested vaccine formulation samples were of water in oil emulsion type.

***b- Emulsion stability***.

The tested vaccine formulation samples were short- and long-term stable for up to 1 year.

***c- Formalin residues***.

The tested vaccine formulation samples matched the 0.01 formalin standard tube.

#### Basic quality controls (QCs) characteristics of the developed and prepared vaccines

***a- Purity testing***.

The examined cultures and microscopic slides showed pure Gram-positive cocci that appeared in long chains.

***b- Sterility testing***.

No evidence of any bacterial contaminants (aerobic or anaerobic) or fungal growth was detected after prolonged incubation of cultured vaccine samples on appropriate media.

***c- Safety testing***.

Safety studies on the prepared vaccines proved their safety where no deaths and no behavioral or physical (local or general) abnormal reactions reaction were observed in mice, rabbits, and foals injected with any of the four vaccinal preparations. The injected foals didn’t show any adverse post-vaccinal reaction either locally at the site of injection or systemically through a monthly observation period from the time of vaccination up to 6 months post-vaccination.

### Immunizing potential and potency of the developed and prepared vaccines

#### Immunization of isogenic BALB/c mice

**Fig. 1:**
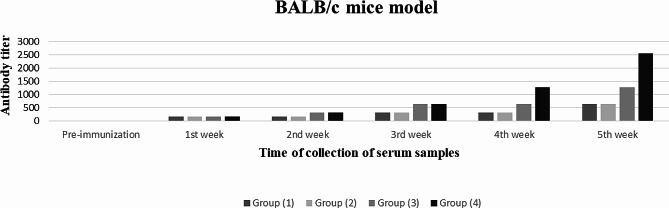
Graphical presentation of the antibody titer in case of developed vaccine formulations

The result of the measurement of the *S. equi*-specific antibodies using PHA is demonstrated in Fig. 1; Table 3. In BALB/c mice immunized with *S. equi*-adjuvanted with either MONTANIDE GEL (Group 1) or MONTANIDE ISA-70 adjuvants (Group 2), a PHA antibody titer of 160 was recorded after two weeks from the first immunization dose. While a PHA titer of 320 was recorded in BALB/c mice immunized with the combined vaccine adjuvanted with MONTANIDE GEL (Group 3) or MONTANIDE ISA-70 adjuvants (Group 4), after two weeks from the first immunization dose. One week after the second immunization dose of *S. equi* adjuvanted with either MONTANIDE GEL (Group 1) or MONTANIDE ISA-70 adjuvants (Group 2) a titer of 320 was recorded in serum from both groups. Higher PHA antibody titers, however, were measured at the end of the 3rd week after the 2nd immunization, in mice immunized with the combined vaccines where an antibody titer of 640 was recorded in mice immunized with the combined vaccine adjuvanted with MONTANIDE GEL (Group 3), and a titer of 2560 was measured in serum samples in mice from (Group 4) immunized with the combined vaccine adjuvanted with MONTANIDE ISA-70 adjuvant. From the obtained results no significant differences were reported in the immune response developed against the four vaccine formulations. However, the *S. equi* combined vaccine adjuvanted with MONTANIDE ISA-70 gave the highest PHA antibody titer.

**Table 3:** *S. equi* -specific antibody titers in pooled serum samples from the four groups of the immunized BALB/c mice as measured by PHA test

Time of collection of serum samples	Antibody titer
**Pre-immunization (Day zero)**	
♣ Group (1): *S. equi* adjuvanted with MONTANIDE GEL	Negative
♣ Group (2): *S. equi* adjuvanted with MONTANIDE ISA-70	Negative
♣ Group (3): Combined vaccine adjuvanted with MONTANIDE GEL	Negative
♣ Group (4): Combined vaccine adjuvanted with MONTANIDE ISA-70	Negative
**1st immunization**	
**One-week post-immunization (Day 7)**	
♣ Group (1): *S. equi* adjuvanted with MONTANIDE GEL	160
♣ Group (2): *S. equi* adjuvanted with MONTANIDE ISA-70	160
♣ Group (3): Combined vaccine adjuvanted with MONTANIDE GEL	160
♣ Group (4): Combined vaccine adjuvanted with MONTANIDE ISA-70	160
**Two-weeks post-immunization (Day 14)**	
♣ Group (1): *S. equi* adjuvanted with MONTANIDE GEL	160
♣ Group (2): *S. equi* adjuvanted with MONTANIDE ISA-70	160
♣ Group (3): Combined vaccine adjuvanted with MONTANIDE GEL	320
♣ Group (4): Combined vaccine adjuvanted with MONTANIDE ISA-70	320
**2nd immunization (1st booster)**	
**One-week post-immunization (Day 21)**	
♣ Group (1): *S. equi* adjuvanted with MONTANIDE GEL	320
♣ Group (2): *S. equi* adjuvanted with MONTANIDE ISA-70	320
♣ Group (3): Combined vaccine adjuvanted with MONTANIDE GEL	640
♣ Group (4): Combined vaccine adjuvanted with MONTANIDE ISA-70	640
**Two-weeks post-immunization (Day 28)**	
♣ Group (1): *S. equi* adjuvanted with MONTANIDE GEL	320
♣ Group (2): *S. equi* adjuvanted with MONTANIDE ISA-70	320
♣ Group (3): Combined vaccine adjuvanted with MONTANIDE GEL	640
♣ Group (4): Combined vaccine adjuvanted with MONTANIDE ISA-70	1280
**Three-weeks post-immunization (Day 35)**	
♣ Group (1): *S. equi* adjuvanted with MONTANIDE GEL	640
♣ Group (2): *S. equi* adjuvanted with MONTANIDE ISA-70	640
♣ Group (3): Combined vaccine adjuvanted with MONTANIDE GEL	1280
♣ Group (4): Combined vaccine adjuvanted with MONTANIDE ISA-70	2560

#### Immunization of white New Zealand rabbits

Using the passive hemagglutination test a titer of 160 was recorded in pooled serum samples from rabbit groups immunized with the four formulations of *S. equi* vaccines after two weeks from the first immunization dose. After one week from the second immunization dose, the titer increased to 320 in all the immunized 4 groups. One week after the third booster dose the *S. equi*-specific antibodies’ titer increased gradually till it reached 640 in all groups. When the antibody titers were measured after one month from the third dose the titers remained at 640 while in (Group 4) immunized with the combined vaccine and adjuvanted with MONTANIDE ISA-70, one log increase in the antibody titer was recorded to be 1280 Table 4.

**Table 4:** *S. equi*-specific antibody titer as measured by PHA in sera of rabbits immunized with the four formulations of *S. equi* vaccine using the M protein of *S. equi* as sensitizing antigen

Time of collection of serum samples	Antibody titer
**Pre-immunization (Day zero)**	
Group (1): *S. equi* adjuvanted with MONTANIDE GEL	Negative
Group (2): *S. equi* adjuvanted with MONTANIDE ISA-70	Negative
Group (3): Combined vaccine adjuvanted with MONTANIDE GEL	Negative
Group (4): Combined vaccine adjuvanted with MONTANIDE ISA-70	Negative
**1st immunization**	
**One-week post-immunization (Day 7)**	
Group (1): *S. equi* adjuvanted with MONTANIDE GEL	160
Group (2): *S. equi* adjuvanted with MONTANIDE ISA-70	160
Group (3): Combined vaccine adjuvanted with MONTANIDE GEL	160
Group (4): Combined vaccine adjuvanted with MONTANIDE ISA-70	160
**Two-weeks post-immunization (Day 14)**	
Group (1): *S. equi* adjuvanted with MONTANIDE GEL	160
Group (2): *S. equi* adjuvanted with MONTANIDE ISA-70	160
Group (3): Combined vaccine adjuvanted with MONTANIDE GEL	160
Group (4): Combined vaccine adjuvanted with MONTANIDE ISA-70	160
**2nd immunization (1st booster)**	
**One-week post-immunization (Day 21)**	
Group (1): *S. equi* adjuvanted with MONTANIDE GEL	320
Group (2): *S. equi* adjuvanted with MONTANIDE ISA-70	320
Group (3): Combined vaccine adjuvanted with MONTANIDE GEL	320
Group (4): Combined vaccine adjuvanted with MONTANIDE ISA-70	320
**Two-weeks post-immunization (Day 28)**	
Group (1): *S. equi* adjuvanted with MONTANIDE GEL	320
Group (2): *S. equi* adjuvanted with MONTANIDE ISA-70	320
Group (3): Combined vaccine adjuvanted with MONTANIDE GEL	320
Group (4): Combined vaccine adjuvanted with MONTANIDE ISA-70	320
**3rd immunization (2nd booster)**	
**One-week post-immunization (Day 35)**	
Group (1): *S. equi* adjuvanted with MONTANIDE GEL	640
Group (2): *S. equi* adjuvanted with MONTANIDE ISA-70	640
Group (3): Combined vaccine adjuvanted with MONTANIDE GEL	640
Group (4): Combined vaccine adjuvanted with MONTANIDE ISA-70	640
**Two-weeks post-immunization (Day 42)**	
Group (1): *S. equi* adjuvanted with MONTANIDE GEL	640
Group (2): *S. equi* adjuvanted with MONTANIDE ISA-70	640
Group (3): Combined vaccine adjuvanted with MONTANIDE GEL	640
Group (4): Combined vaccine adjuvanted with MONTANIDE ISA-70	1280
**Three-weeks post-Immunization (Day 49)**	
Group (1): *S. equi* adjuvanted with MONTANIDE GEL	640
Group (2): *S. equi* adjuvanted with MONTANIDE ISA-70	640
Group (3): Combined vaccine adjuvanted with MONTANIDE GEL	640
Group (4): Combined vaccine adjuvanted with MONTANIDE ISA-70	1280

### Protective efficacy of the developed and prepared vaccines

#### Laboratory challenge in immunized BALB/c mice with *S. equi*

In the negative control three non-immunized, the first death of mice occurred 24 h after the challenge, and the mortality rate reached 100% within 48 h, while among the immunized mice, 5 of 12 mice died in case of the monovalent vaccines (58% protective efficacy), 4 of 12 mice died in case of the MONTANIDE GEL adjuvanted combined vaccine (67% protective efficacy), and only 3 out of 12 mice died in case of the MONTANIDE ISA-70 adjuvanted combined vaccine, following the challenge with *S. equi* (75% protective efficacy). More specifically, the deaths occurred between the 3rd and 5th day post-challenge. *S. equi* was isolated from the internal organs of dead animals.

#### Field challenge in unvaccinated/exposed foals

The obtained results are shown in Tables 5 and 6. From the measured optical density (OD) of the undiluted negative control sample (Sample 1) and from undiluted samples that were collected from the foals before immunization (Samples 2, 3, and 4), the cut-off value (COV) was calculated as follows; The COV = X ± (SD × 2) = 0. 283 ± (0.051 × 2) = 0.385. Accordingly, any sample that gives an OD reading equal to or more than 0.385 is considered positive.

**Table 5:** Results of indirect ELISA on serum samples from immunized foals expressed as OD using prepared *seM*-like protein as coating antigen (measured at 405 nm). The measured OD of serum sample (1) from the control negative foal and serum samples (2, 3, and 4) collected before immunization from the group to be vaccinated (three foals) were used to calculate the COV that was equal to 0.385

Serum samples from foals	Serum dilution									
	1/200	1/400	1/800	1/1600	1/3200	1/6400	1/12,800		1/25,600	
		**OD from Control negative**								
**Sample (1)**	0.353	0.260	0.249	0.220	0.199	0.198		0.150		0.109
		**OD from foals to be vaccinated (pre-vaccination) (Day 0)**								
**Sample (2)**	-ve	0.350	0.317	0.372	0.298	0.250		0.197		0.164
**Sample (3)**	0.232	0.210	0.170	0.174	0.181	0.151		0.139		0.164
**Sample (4)**	0.263	0.540	0.327	0.372	0.307	0.216		0.194		0.216
		**Control-positive (infected foals)**								
**Sample (5)**	0.826	0.790	0.574	0.466	0.595	0.572		0.196		0.145
**Sample (6)**	0.562	0.630	0.556	0.510	0.540	0.645		-ve		-ve
		**Two weeks after the first immunization dose (Day 14)**								
**Sample (7)**	0.710	0.580	0.775	0.450	0.176	0.278		0.282		0.287
**Sample (8)**	0.605	0.660	0.674	0.386	0.204	0.243		0.311		0.242
**Sample (9)**	0.657	0.640	0.667	0.522	0.391	-ve		-ve		-ve
		**Two weeks after the second immunization (1st booster) dose (Day 28)**								
**Sample (10)**	0.875	0.760	0.604	0.519	0.388	0.391		0.311		0.266
**Sample (11)**	0.618	0.580	0.623	0.533	0.312	0.393		0.324		0.359
**Sample (12)**	0.517	0.560	0.502	0.464	0.366	0.412		0.359		0.290

**Table 6:** Results of indirect ELISA on serum samples from foals immunized with *S. equi* combined vaccine adjuvanted with MONTANIDE ISA-70 using the prepared *seM* protein of *S. equi* as coating antigen (measured at 405 nm)

Serum samples from vaccinated foals	Results
**Sample (1)** Control Negative	The measured OD from samples (2–6) was used to calculate the COV that was equal to 0.385
**Samples (2,3,4)** Before vaccination **(Day 0)**	
**Control-positive (infected foals)**	
**Sample 5**	Ab titer = 6400 ELISA unit/ml
**Sample 6**	Ab titer = 6400 ELISA unit/ml
**Two weeks after the first immunization dose (Day 14)**	
**Sample 7**	Ab titer = 1600 ELISA unit/ml
**Sample 8**	Ab titer = 1600 ELISA unit/ml
**Sample 9**	Ab titer = 3200 ELISA unit/ml
**Two weeks after the second immunization (1st booster) dose (Day 28)**	
**Sample 10**	Ab titer = 6400 ELISA unit/ml
**Sample 11**	Ab titer = 6400 ELISA unit/ml
**Sample 12**	Ab titer = 6400 ELISA unit/ml

## Discussion

Acute infectious upper respiratory tract disease known as “strangles” primarily affects young horses and is characterized by lymphadenitis of the submandibular and retropharyngeal lymph nodes as well as rhino-pharyngitis. Due to the extended duration of recovery, quarantine, and treatment required, strangles epidemics in farms and stables can linger for months or even years, having a significant negative economic impact [40–42].

Fever is usually the first clinical sign of strangles, followed by a serous to mucoid nasal discharge that eventually turns purulent. The production of pyrogenic exotoxins is probably what causes fever induction [43], and peptidoglycan is also thought to be pyrogenic since it causes leukocytes to release pyrogenic pro-inflammatory cytokines [44]. In the lymph nodes of the head and neck, the submandibular and retropharyngeal lymph nodes are frequently affected, *S. equi* invasion of local lymph nodes and the ensuing inflammation cause swelling and abscess formation. The disease is known by its English term, “strangles,” since severe swelling of the local lymph nodes can result in breathing problems and even death from asphyxiation. A lot of mucopurulent nasal discharge could result from ruptured abscessed lymph nodes that flow into the pharynx. Additionally, drainage into guttural pouches can happen from retropharyngeal lymph nodes [10].

The inspected horses’ nasal swabs and abscess contents were inoculated onto Edward’s medium plates. The isolates that were found were β-hemolytic streptococci that were Gram-positive and belonged to Lancefield group C. On Edward’s blood agar, it usually formed big mucoid colonies with a broad zone of β-hemolysis and was well encapsulated. The incapacity of *S. equi* to ferment sorbitol and lactose distinguished it from other group C streptococci [45]. Hyaluronic acid (HA) is the reason why *S. equi* colonies have a mucoid look. A PCR confirmatory step has been done as PCR has been extensively used for the detection of *S. equi* since the species-specific M protein *(seM*) sequence in this organism was reported by [26, 27]. *S. equi* PCR targeting this specific M protein has been proven to be quite specific and there is also no evidence that a *seM*-like protein is expressed by *S. zooepidemicus* or other species [10].

To stimulate a systemic immune response, most immunization regimens against strangles have relied on intramuscular injections of vaccines. Nevertheless, most of these commercially available vaccinations have only been partially successful in lowering the sickness in 60–70% of the horses that the organism has challenged. Furthermore, unpleasant effects from intramuscular injections can last for up to a week and frequently result in fever, malaise, and painful muscles [10]. Despite being inactivated and intended for intramuscular injection, the recently developed vaccines demonstrated a higher level of protective efficacy, reaching 75%. Furthermore, no severe adverse or harmful post-vaccinal reactions were observed during the vaccine injection process or the two-week post-vaccination observation period among the vaccinated foals included in the safety testing or the vaccinated foals included in the field challenge testing.

As [46] suggests, the low immunogenicity of commercial vaccines and limited antigenic connections between field strains and vaccines may be responsible for the low protection rates observed in the herds under study. Furthermore, the protection produced by inactivated vaccines, which use subunits as antigens, is typically insufficient to fend off a pathogenic strain challenge [47]. In this investigation, four new formulations of an inactivated *S. equi* vaccine were introduced. Two field strangles-incriminated streptococci (*S. equi* and *S. zooepidemicus*) were used to manufacture these vaccines, together with two recently added adjuvants (MONTANIDE GEL and MONTANIDE ISA-70) that underwent testing and evaluation. A series of oil/surfactant-based adjuvants known as MONTANIDE incomplete adjuvants is made up of different surfactants combined with one of three mineral oils: one that can be metabolized, one that cannot, or a mixture of the two oils [48]. Vaccine compositions containing MONTANIDE elicit strong and long-lasting protection. MONTANIDE emulsions are stable and simple to inject, and they have a higher immunopotentiation capacity and fewer side effects than traditional oil emulsions [49].

Because inactivated vaccines frequently have low immunogenicity, the right adjuvants must be added to boost the immune system’s response to the antigens. Adjuvants may also reduce the amount of antigen or vaccinations needed to produce a protective immune response, which could lead to a decrease in the price of vaccines and an increase in their accessibility. Water-in-oil (W/O) emulsion vaccines are used in many nations across the world. MONTANIDE ISA-70 is a mineral-oil-based adjuvant that is made up of a naturally metabolizable oil and a highly refined emulsifier from the mannide monooleate family in an oily solution. Oil-adjuvanted vaccine formulations elicit greater and more sustained immune responses than other adjuvanted vaccine formulations [50].

Serum samples were taken from the inoculated animals before and at various time points after the vaccination to measure the level of the vaccine-induced *S. equi*-specific antibodies because these antibodies appear to have a significant protective effect [51]. According to previous studies, humoral immunity plays a major role in protecting horses from *S. equi* infections, and there may be a correlation between the level of immunity and antibody production [51]. Additionally [52], showed that most of the foals had anti-M-protein PHA titers ranging from 320 to 2560, making them immune to strangles because of vaccination or prior infection. In this study, mice received two doses of each of the four *S. equi* vaccine formulations and exhibited anti-*S. equi*-specific antibody titers of 320, 640, and higher, as determined by the PHA test, are deemed protective against strangles. The immunological response generated against the four vaccine formulations did not differ significantly according to the obtained results After the second dose of immunization, the *S. equi* combination vaccine adjuvanted with MONTANIDE ISA-70 produced the highest PHA antibody titer of 1/2560.

Following receiving the second and third doses of the produced vaccines, rabbits showed anti-*S. equi* antibody titers of 1/320 and 1/640, respectively, one week following vaccination. These antibody concentrations are thought to be defensive. Additionally, the antibody titer of rabbits inoculated with the MONTANIDE ISA-70 adjuvanted, and combination vaccination was 1/1280 and showed a log increase in comparison to those immunized with the monovalent vaccines. The cross-reactivity of sera from a horse convalescent from strangles with several different *S. equi* isolates and the lack of variation in Hind III restriction patterns between different *S. equi* isolates on southern blot analysis using a *seM* gene probe, according to [53], led to the assumption that *seM* was highly homogeneous. In this work, the monovalent vaccination adjuvanted with either MONTANIDE ISA-70 or MONTANIDE GEL produced a lower antibody titer against the M protein of *S. equi* than the inactivated combination vaccine adjuvanted with MONTANIDE ISA-70 in white New Zealand rabbits and isogenic BALB/c mice. The inclusion of both strains in a single vaccination is thought to be beneficial since a combination vaccine produces a high titer of antibodies against both subspecies. No mortality or stress-induced pathological conditions were detected in animals inoculated with the two adjuvants under test, and the antibody production against the strangles pathogen was boosted without any indication of a local or general reaction. Nonetheless, it was noted that the combination vaccination adjuvanted with MONTANIDE ISA-70 mineral oil-based adjuvant yielded the highest titer.

Indirect ELISA has been previously used by [54] to detect antibodies against strangles. The increased virulence of *S. equi* compared to its evolutionary ancestor, *S. zooepidemicus*, has been attributed to the production of a novel M-like protein, *seM* [55, 56]. The anti-phagocytic effect of *seM* is comparable to that of the M proteins of group A streptococci, as it actively binds fibrinogen and IgG and prevents the deposition of C3b on the bacterial surface [57]. Strong opsonic characteristics are exhibited by antibodies produced against *seM*, which is highly immunogenic [26, 56]. Furthermore, the homology between it and its *S. zooepidemicus* homolog is extremely low (23%). In the current investigation, 325 mg/dl of *seM* antigen was produced and utilized as a coating antigen in ELISA. The analyzed sera’s antibody titer against *seM* protein was measured using indirect ELISA. Additionally, foals’ serological responses developed gradually and were observed over a long period following immunization. The foals that received the immunization had their maximum ELISA antibody levels measured 28 days after the shot at a dilution of 1/6400.

## Conclusion

The newly developed vaccines were used in the study’s laboratory and field challenge tests. It’s important to note that animals immunized with the inactivated combined vaccine adjuvanted with MONTANIDE ISA-70 were found to be 75% protected against challenges with virulent *S. equi* strains. The performance of the newly designed vaccine is highly promising and is competitive and promisingly comparable to that of the presently available strangles vaccines, according to the results of the current experiment. After two doses (prime and booster doses), the recently developed vaccine produced strong specific antibody titers under field conditions that persisted for 4–6 months, demonstrating the vaccine’s capacity to offer field-clinical protection. Although the differences between the Ab titers produced against the four developed and prepared vaccine formulations weren’t significant, the study’s findings proved the significance and the protective efficacy of the inactivated combined vaccination adjuvanted with MONTANIDE ISA-70 (W/O) emulsion, which is based on mineral oil and contains combined locally isolated strains of *S. equi* and *S. zooepidemicus* from Egypt. Overall, the results obtained strongly imply that the recently developed combined and inactivated vaccine adjuvanted with MONTANIDE ISA-70 may prove to be a very effective strategic confrontation approach for the mitigation and control of *S. equi* infections, especially in countries and regions where strangles is an epidemic.

## Data Availability

All data generated or analyzed during this study are included in this published article. The data used and/or analyzed during the current study are available from the corresponding author upon reasonable request.
